# Recent Progress in Fused-Ring Based Nonfullerene Acceptors for Polymer Solar Cells

**DOI:** 10.3389/fchem.2018.00404

**Published:** 2018-09-25

**Authors:** Chaohua Cui

**Affiliations:** Laboratory of Advanced Optoelectronic Materials, College of Chemistry, Chemical Engineering and Materials Science, Soochow University, Suzhou, China

**Keywords:** polymer solar cells, nonfullerene acceptor, molecular design, power conversion efficiency, energy levels

## Abstract

The progress of bulk-heterojunction (BHJ) polymer solar cells (PSCs) is closely related to the innovation of photoactive materials (donor and acceptor materials), interface engineering, and device optimization. Especially, the development of the photoactive materials dominates the research filed in the past decades. Photoactive materials are basically classified as p-type organic semiconductor donor (D) and an n-type organic semiconductor acceptor (A). In the past two decades, fullerene derivatives are the dominant acceptors for high efficiency PSCs. Nevertheless, the limited absorption and challenging structural tunability of fullerenes hinder further improve the efficiency of PSCs. Encouragingly, the recent progresses of fused-ring based A-D-A type nonfullerene acceptors exhibit great potential in enhancing the photovoltaic performance of devices, driving the power conversion efficiency to over 13%. Such kind of nonfullerene acceptors is usually based on indacenodithiophene (IDT) or its extending backbone core and end-caped with strong electron-withdrawing group. Owing to the strong push-pulling effects, the acceptors possess strong absorption in the visible-NIR region and low-lying HOMO (highest occupied molecular orbital) level, which can realize both high open-circuit voltage and short-circuit current density of the devices. Moreover, the photo-electronic and aggregative properties of the acceptors can be flexibly manipulated via structural design. Many strategies have been successfully employed to tune the energy levels, absorption features, and aggregation properties of the fused-ring based acceptors. In this review, we will summarize the recent progress in developing highly efficient fused-ring based nonfullerene acceptors. We will mainly focus our discussion on the correlating factors of molecular structures to their absorption, molecular energy levels, and photovoltaic performance. It is envisioned that an analysis of the relationship between molecular structures and photovoltaic properties would contribute to a better understanding of this kind of acceptors for high-efficiency PSCs.

## Introduction

**Figure 1:**
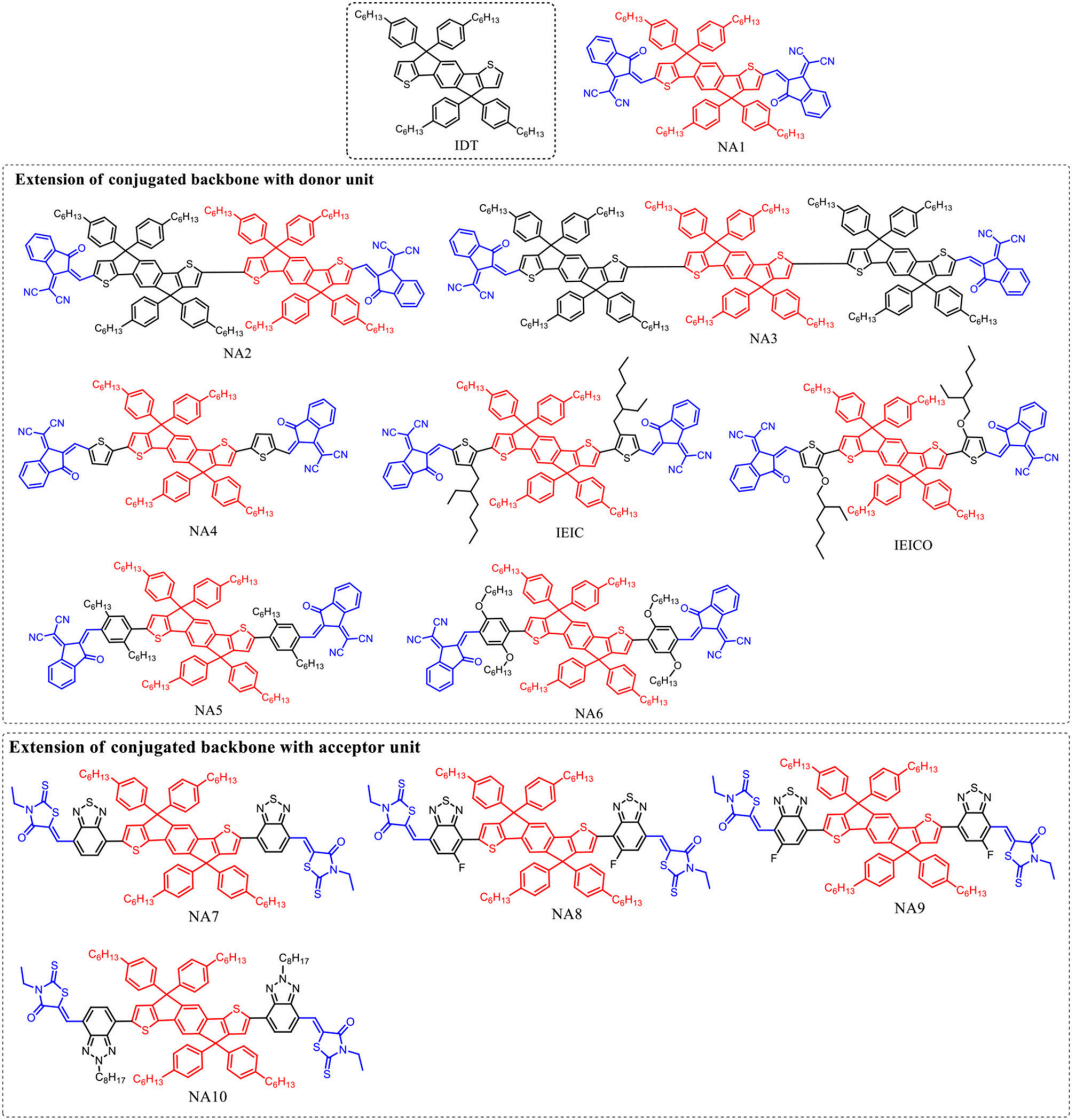
IDT core based nonfullerene acceptors with different π conjugation extension.

Typically, bulk-heterojunction (BHJ) polymer solar cells (PSCs) are composed of a photoactive layer sandwiched between a transparent anode and a low work function metal cathode (Li, 2012; Li et al., 2012; Nielsen et al., 2012; Chen et al., 2013; Heeger, 2013; Janssen and Nelson, 2013; Zhan et al., 2015; Elumalai and Uddin, 2016; Zhan and Yao, 2016; Zhang et al., 2017). The PCE of PSC is proportional to open-circuit voltage (*V*_oc_), short-circuit current density (*J*_sc_), and fill factor (FF). The progress of PSCs is closely related to the innovation of photoactive materials (donor and acceptor materials) (He and Li, 2011; Li, 2013; Cui et al., 2014, 2016; Ye et al., 2014; Lu et al., 2015; Cui and Wong, 2016; Cui Y. et al., 2017; Hu et al., 2017; Lopez et al., 2017; Osaka and Takimiya, 2017; Zou et al., 2017; Gupta et al., 2018; Liu et al., 2018; Sun et al., 2018), interface engineering (He et al., 2012; Duan et al., 2013; Chueh et al., 2015; Wang et al., 2015; Chen et al., 2016; Street, 2016), and device optimization (Ameri et al., 2009, 2013; Meillaud et al., 2015; Zhao et al., 2018). Especially, the development of PSCs is always accompanied by photoactive material innovations. As the key component, photoactive materials are basically classified as a p-type organic semiconductor donor (D) and an n-type organic semiconductor acceptor (A). Due to the unique advantages of strong electron-accepting and high electron-transport capabilities, fullerene derivatives were predominately used as the acceptor in PSCs in the past two decades, driving the power conversion efficiency (PCE) of PSCs to 11–12% (Liu et al., 2014; Zhao J. et al., 2016). Nevertheless, fullerenes based acceptors show critical shortcomings of weak absorption and limited structural modification, hindering further improve photovoltaic performance of devices. To overcome these obstacles of fullerenes based acceptors, many efforts have been devoted to developing new kind of nonfullerene acceptor materials (Hendriks et al., 2014; Cheng et al., 2018; Hou et al., 2018; Yan et al., 2018). Very recently, A-D-A conjugated fused-ring molecules based on indacenodithiophene (IDT, Figure 1) or DTIDT unit (Figure **4**) were reported as excellent nonfullerene acceptors for high performance PSCs, leading the PCE of device to over 13% (Wang et al., 2016; Li S. et al., 2018). Very recently, the PCEs of nonfullerene based PSCs have been driven to a milestone of over 14% (Li S. et al., 2018; Zhang et al., 2018). Unlike fullerene derivatives, fused-ring based nonfullerene acceptor materials offer many molecular design strategies to tune their optoelectronic properties and thus photovoltaic performance. In this review, we will provide some representative cases of molecular manipulation on IDT and DTIDT based nonfullerene acceptors to fine-tune the physicochemical and photovoltaic properties. We hope that this review article would contribute to a better understanding of the design strategies of high performance fused-ring based acceptors for efficient nonfullerene PSCs.

## IDT based fused-ring acceptors

IDT unit (Figure 1) which features phenylene ring fused to thiophene was firstly reported by Wong in 2006 (Wong et al., 2006) Such fused rings structure is beneficial to forming effective interchain π-π overlap and enhance the rigidity of the molecular backbone as well as the degree of conjugation. Zhan et al. innovatively used IDT as central core to develop an A-D-A (A = acceptor, D = donor) type acceptor material (NA1) with 2-(3-oxo-2,3-dihydroinden-1-ylidene)-malononitrile as terminal acceptor unit (Lin et al., 2016a). NA1 showed promising energy levels and absorption spectrum as acceptor material for PSCs. By using NA1 as acceptor and PDBT-T1 as donor to fabricate PSC device, a high PCE of 7.39% was obtained, with *V*_oc_ = 0.92 V, *J*_sc_ = 13.39 mA cm^−2^, and FF = 0.60 (Table **2**) (Lin et al., 2016a). Molecular optimization based on NA1 greatly affects the photovoltaic performance. In the following, we will discuss the molecular design strategies including extension of conjugated backbone, substituted side chains, and end-capped group were conducted on NA1.

### Extension of conjugated backbone with donor unit

The *V*_oc_ of PSCs device is tightly correlated with the energy level difference between the HOMO of the donor and the LUMO of the acceptor. Therefore, high LUMO level of acceptor material is essential for achieving high *V*_oc_ value. In D-A conjugated molecular system, the donor unit mainly determines the HOMO level. In other words, the optical bandgap (*E*_g_) of D-A based molecules can be tuned by incorporating donor unit as conjugated extension while maintaining the similar LUMO level. For example, Zhan et al. employed one and two IDT units as conjugated extension block to develop two molecules NA2 and NA3 (Lin et al., 2016a). Due to the longer conjugated backbone, the absorption profiles of NA2 and NA3 are effectively red-shifted compared to NA1, and NA3 possesses the lowest *E*_g_ of 1.53 eV (Table 1). On the other hand, NA2 and NA3 showed up-shifted HOMO levels while similar LUMO levels. Due to the weaker molecular π-π stacking compared to NA1, the NA2 based device exhibited a low PCE of 2.58%, while no photovoltaic response was observed from the NA3 based device. In comparison with NA1, NA4 with thiophene units as π-bridge for conjugated extension showed slightly red-shifted absorption spectrum (*E*_g_ = 1.55 eV), up-shifted HOMO level of −5.42 eV, and similar LUMO level of −3.85 eV (Bai et al., 2015a). The device based on PBDTTT-C-T:NA4 exhibited a PCE of 3.93%, with a *V*_oc_ of 0.90 V, *J*_sc_ of 8.33 mA cm^−2^, and FF of 0.523 (Table 2). Presumably owing to the conjugated twists, the *E*_g_ was increased to 1.57 eV when attaching 2-ethylhexyl chains in thiophene π-bridge of NA1 (IEIC, Figure 1) (Lin et al., 2015b). By using IEIC as acceptor and PTB7-Th as donor to fabricate device, a promising PCE of 6.31% was obtained, with *V*_oc_ = 0.97 V, *J*_sc_ = 13.55 mA cm^−2^, and FF = 0.48 (Table 2). To reduce the *E*_g_ of IEIC, Hou et al. replaced the 2-ethylhexyl chains of IEIC with alkoxy chains to develop a new acceptor IEICO (Yao et al., 2016). Attributing to the strong electron-donating ability of alkoxy chains, IEICO exhibited a lower *E*_g_ of 1.34 eV than IEIC. Relative to IEIC, the density functional theory calculation result suggests that the introduction of alkoxy chains effectively up-shifted the HOMO level (~0.19 eV) while maintaining the similar LUMO level (0.01 eV lower than IEIC). The PSCs using IEICO as acceptor yielded a high PCE of 8.4%, with a *V*_oc_ of 0.82 V and a *J*_sc_ of 17.7 mA cm^−2^, while the control device with IEIC as acceptor exhibited a much lower PCE of 4.9% (Table 2) (Yao et al., 2016). In comparison with IEIC-based PSCs, the higher *J*_sc_ value of IEICO-based device should be resulted from the much broader photo-response spectrum with higher external quantum efficiency. Bo et al. used bis(alkoxy)-substituted or dialkyl-substituted benzene ring as π bridge for conjugated extension to develop two molecules NA5 and NA6 (Figure 1) (Liu Y. et al., 2017). Benefiting from the non-covalent S···O interaction locks, NA6 exhibited better planarity and broader absorption spectrum than NA5, with a lower *E*_g_ of 1.63 eV (Table 1). In addition, NA6 with locked conformation exhibited a higher quantum yield, which can effectively suppress the non-radiative energy loss and afford higher *V*_oc_ for devices. The device based on NA6 realized a promising PCE of 9.60%, with *V*_oc_ = 1.01 V, *J*_sc_ = 17.52 mA cm^−2^, and FF = 0.54, while the NA5 based device showed a much lower PCE of 2.3% (Table 2).

**Table 1:** Summary of absorption properties and energy levels of IDT core based nonfullerene acceptors shown in Figure 1.

**Acceptor**	**Bandgap [eV]**	**HOMO [eV]**	**LUMO [eV]**
NA1	1.70	−5.91	−3.83
NA2	1.57	−5.42	−3.80
NA3	1.53	−5.29	−3.79
NA4	1.55	−5.43	−3.85
IEIC	1.57	−5.42	−3.82
IEICO	1.34	−5.32	−3.95
NA5	1.75	−5.55	−3.82
NA6	1.63	−5.51	−3.78
NA7	1.68	−5.52	−3.69
NA8	1.67	−5.64	−3.73
NA9	1.71	−5.67	−3.74
NA10	1.87	−5.46	−3.57

**Table 2:** Summary of photovoltaic properties of the nonfullerene acceptors shown in Figure 1.

**Active layer**	***V*_oc_ [v]**	***J*_sc_ [mA cm^−2^]**	**FF**	**PCE [%]**	**References**
PDBT-T1:NA1	0.92	13.39	0.60	7.39	Lin et al., 2016a
PDBT-T1:NA2	1.02	5.28	0.48	2.58	Lin et al., 2016a
PDBT-T1:NA3	–	–	–	–	Lin et al., 2016a
PBDTTT-C-T:NA4	0.90	8.33	0.523	3.93	Bai et al., 2015b
PTB7-Th:IEIC	0.97	13.55	0.48	6.31	Lin et al., 2015b
PBDTTT-ET:IEICO	0.82	17.7	0.58	8.4	Yao et al., 2016
PBDTTT-ET:IEIC	0.90	11.7	0.47	4.90	Yao et al., 2016
PBDB-T:NA5	0.92	5.63	0.55	2.3	Liu Y. et al., 2017
PBDB-T:NA6	1.01	17.52	0.54	9.60	Liu F. et al., 2017
P3HT:NA7	0.84	8.91	0.681	5.12	Wu et al., 2015
PTzBI:NA8	1.00	11.6	0.623	7.44	Zhong et al., 2017
PTzBI:NA9	0.99	9.4	0.559	5.28	Zhong et al., 2017
J61:NA10	1.24	5.21	0.467	3.02	Tang et al., 2018

As demonstrated above, using electron rich unit as conjugated backbone extension for NA1 is an effective strategy to broaden absorption spectrum, rise up HOMO levels while maintain similar LUMO levels of resulting molecules.

### Extension of conjugated backbone with acceptor unit

Since the LUMO distribution is mainly located at the acceptor unit in D-A conjugated molecular system, using acceptor unit as building block can simultaneously manipulate the LUMO level and *E*_g_ of the molecules. Zhan et al. develop a nonfullerene acceptor NA7 (Figure 1) using benzothiadiazole as π-bridge (Wu et al., 2015). NA7 shows flat backbone configuration which is beneficial for charge transport, and a large dihedral angle between the hexylphenyl group and backbone plane which can prevent the over self-aggregation when blending with P3HT. Due to the relatively high LUMO level of NA7, the device based on P3HT:NA7 exhibited a high *V*_oc_ of 0.84 V, with a high PCE of 5.12% (Table 2) (Wu et al., 2015). Bazan et al. developed two NA7 analogs, NA8 and NA9 (Figure 1), with different positions of the fluorine atom in benzothiadiazole unit (Zhong et al., 2017). Relative to NA7 (*E*_g_ = 1.68 eV), NA8 exhibited a similar *E*_g_ of 1.67 eV, while NA9 showed a slightly larger *E*_g_ of 1.71 eV. The orientations of the fluorine atoms show little influence in the HOMO and LUMO levels but affect the calculated conformational diversity and the electrostatic potential of the molecules. The device based on PTzBI:NA8 exhibited a PCE of 7.44%, higher than that of PTzBI:NA9 based device (PCE = 5.28%). The photovoltaic performance of NA9 based device is poorer than that of NA8 based device, which should be resulted from the less optimal BHJ morphology. Zhou et al. replaced the benzothiadiazole units of NA7 by benzotriazole units to develop NA10 (Figure 1) (Tang et al., 2018). Due to the weaker electron-accepting ability of benzotriazole than benzothiadiazole, NA10 showed a higher LUMO level than NA7. Therefore, the J61:NA10 based device achieved an encouraging *V*_oc_ of 1.24 V, with a PCE of 3.02% (Table 2).

In short, the extension of conjugated backbone with donor or acceptor units will generally broaden absorption spectra and reduce *E*_g_ of the resulting molecules, and the introduction of donor units as π-bridge is more effective to reduce the *E*_g_ than acceptor units. On the other hand, the LUMO levels will be up-shifted when using donor units as π-bridge, while the incorporation of acceptor units will lead to higher LUMO levels.

### Side chains engineering

**Figure 2:**
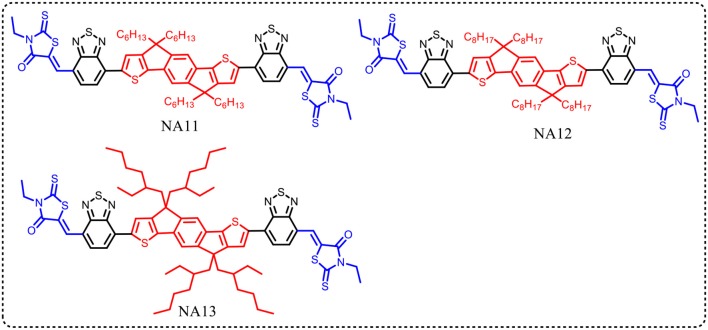
Alkyl chains substituted IDT core based nonfullerene acceptors.

The conjugated side chains substituents on the IDT unit will increase steric hindrance, reduce intermolecular interactions, and prevent over self-aggregation and large phase separation in blend film. Herein, the physicochemical and photovoltaic properties of IDT based acceptors can easily tune via side chains engineering in IDT unit. Zhan et al. developed an acceptor with non-conjugated alkyl chains in IDT unit (NA11, Figure 2) (Jia et al., 2017). NA11 exhibited a nearly flat molecular backbone configuration, with a lower *E*_g_, higher HOMO level, and higher electron mobility than NA7 (Table 3). The device based on PTB7-Th:NA11 yielded a higher PCE of 8.7% than that of PTB7-Th:NA7 based device (Table 4). Furthermore, the NA11 based device exhibited better thermal stability and photo stability in comparison with NA7 based device. McCulloch et al. reported two alkyl chains substituted IDT based nonfullerene acceptors NA12 and NA 13 (Figure 2) (Holliday et al., 2016). NA12 with linear alkyl chains showed a stronger crystallinity and a narrower *E*_g_ relative to NA13 with branched chains, resulting in higher *J*_sc_ and PCE values (Tables 3, 4). In addition, the oxidative stability of these devices is superior to the benchmark P3HT:PC_60_BM device.

**Table 3:** Summary of absorption properties and energy levels of IDT core based nonfullerene acceptors shown in Figure 2.

**Acceptor**	**Bandgap [eV]**	**HOMO [eV]**	**LUMO [eV]**
NA11	1.61	−5.37	−3.67
NA12	1.63	−5.51	−3.88
NA13	1.68	−5.58	−3.90

**Table 4:** Summary of photovoltaic properties of the nonfullerene acceptors shown in Figure 2.

**Active layer**	***V*_oc_ [v]**	***J*_sc_ [mA cm^−2^]**	**FF**	**PCE [%]**	**References**
PTB-Th:NA7	0.99	13.0	0.60	7.7	Jia et al., 2017
PTB-Th:NA11	0.95	15.2	0.60	8.7	Jia et al., 2017
P3HT:NA12	0.72	13.9	0.60	6.30	Holliday et al., 2016
P3HT:NA13	0.76	12.1	0.62	6.00	Holliday et al., 2016

### Effects of end-capped groups

**Figure 3:**
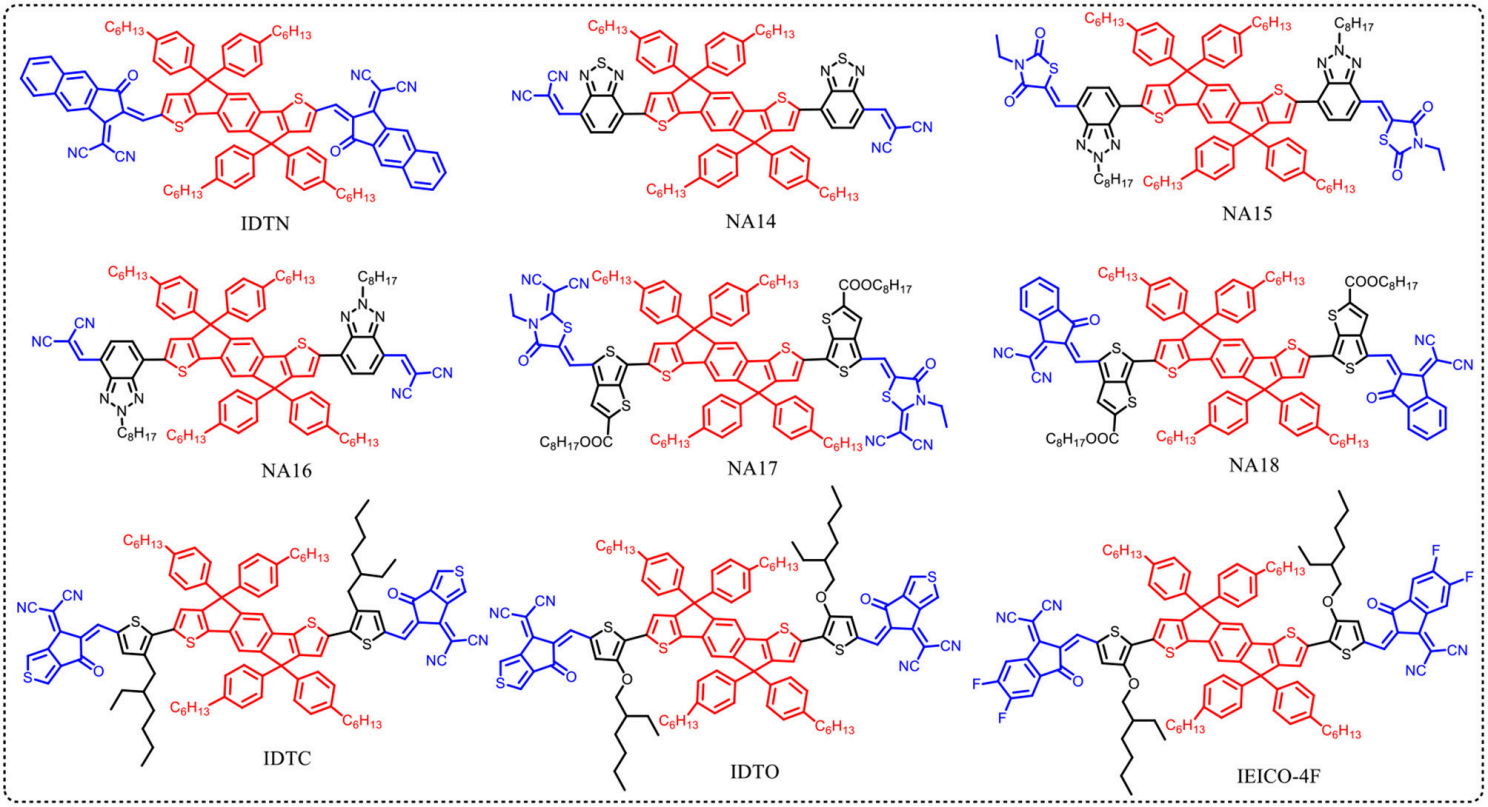
IDT core based nonfullerene acceptors with different end group.

The end-capped groups also affect the optical and electrochemical properties of this kind of nonfullerene acceptors. Hou et al. extended the π-conjugation area of the end group of NA1 to develop a new acceptor IDTN (as shown in Figure 3) (Li S. et al., 2017). The enlarged π-conjugation by phenyl unit in the end group effectively leads to red-shifted absorption and slightly lower LUMO and HOMO levels compared with NA1 (Table 5). Due to the enhanced intermolecular interactions and molecular ordering, IDTN shows a better molecular planarity and higher electron mobility than NA1. Therefore, an outstanding PCE of 12.2% was achieved from the PBDB-TF:IDTN based device, which is significantly higher than that of the NA1 based device (PCE = 7.4%). Zhan et al. used 2-(benzo[c][1,2,5]- thiadiazol-4-ylmethylene)-malononitrile as end-capped groups to develop a new acceptor NA14 (Figure 3) (Bai et al., 2015b). Relative to NA7, the stronger electron-withdrawing ability of end-capped units of NA14 leads to a lower *E*_g_ of 1.60 eV, lower LUMO of −3.8 eV, and lower HOMO of −5.6 eV (Table 5). The PBDTTT-C-T:NA14 based device afforded a relatively high PCE of 4.26%. Zhou et al. systematically engineered the end-capped units of three nonfullerene acceptors to carefully tune the driving force for high *V*_oc_ and *J*_sc_ values (Tang et al., 2018). With the increase of the electron-withdrawing ability of the end-capped units from NA15, NA10, to NA16, the LUMO levels and *E*_g_ simultaneously decrease (Table 5). By fine-tune the LUMO level of acceptor via end-capped unit, NA16 exhibited sufficient energy offset with J61 for efficient charge generation. The device based on J61:NA16 obtained a high PCE of 8.25%, with a high *V*_oc_ of 1.15 V (Table 6). Zhu et al. developed two thieno[3,4-b]thiophene-based acceptor, NA17 and NA18, with different end-capped groups (Figure 3) (Liu et al., 2016; Liu F. et al., 2017) NA17 exhibited an *E*_g_ of 1.54 eV, HOMO level of −5.50 eV, and LUMO level of −3.63 eV. Relative to NA 17, NA18 with stronger electron-withdrawing terminal group possesses lower *E*_g_ of 1.32 eV and deeper LUMO of −3.90 (Table 5). Device based on PTB7-Th:NA17 yielded a high PCE of 10.07%, with *V*_oc_ = 0.87 V, *J*_sc_ = 16.48 mA cm^−2^, and FF = 0.70 (Table 6). Attributing to the deeper LUMO and broader absorption spectrum of NA18, PTB7-Th:NA18 based device exhibited a lower *V*_oc_ of 0.73 V and a higher *J*_sc_ of 16.48 mA cm^−2^, leading to a promising PCE of 9.58% (Table 6). Yang et al. changed the end-capped unit of IEIC and IEICO to develop two analogs IDTC and IDTO (Figure 3) (Luo et al., 2017). Unexpectedly, IDTO showed slightly blue-shifted absorption range relative to IDTC. Nevertheless, the introduction of alkoxy groups effectively improved the intermolecular interactions and up-shifted the LUMO level of IDTO. The device based on PBDB-T:IDTC exhibited a PCE of 9.35%, with a *V*_oc_ of 0.917 V, while the PBDB-T:IDTO based device showed a higher PCE of 10.02% and a higher *V*_oc_ of 0.943 V (Table 6). Hou et al. introduced fluorine atoms onto the end group of IEICO (IEICO-4F, Figure 3) to enhance the intramolecular charge transfer effect. IEICO-4F showed lower *E*g of 1.24 eV and higher LUMO level of −4.19 eV than IEICO. Using IEICO-4F as acceptor, PBDTTT-EFT or J52 as donor, high *J*_sc_ values over 20 mA cm^−2^ were both recorded in the corresponding devices (Table 6).

**Table 5:** Summary of absorption properties and energy levels of IDT core based nonfullerene acceptors shown in Figure 3.

**Acceptor**	**Bandgap [eV]**	**HOMO [eV]**	**LUMO [eV]**
NA1	1.70	−5.81	−3.94
IDTN	1.59	−5.79	−3.98
NA14	1.60	−5.6	−3.8
NA15	2.00	−5.43	−3.46
NA16	1.76	−5.49	−3.61
NA17	1.54	−5.50	−3.63
NA18	1.32	−5.50	−3.90
IDTC	1.51	−5.57	−3.96
IDTO	1.53	−5.52	−3.84
IEICO-4F	1.24	−5.44	−4.19

**Table 6:** Summary of photovoltaic properties of the nonfullerene acceptors shown in Figure 3.

**Active layer**	***V*_oc_ [v]**	***J*_sc_ [mA cm^−2^]**	**FF**	**PCE [%]**	**References**
PBDB-TF:IDTN	0.946	16.58	0.78	12.2	Li S. et al., 2017
PBDB-TF:NA1	0.993	13.01	0.57	7.4	Li S. et al., 2017
PBDTTT-C-T:NA14	0.766	10.10	0.551	4.26	Bai et al., 2015b
J61:NA15	1.29	0.84	0.239	0.26	Tang et al., 2018
J61:NA16	1.15	10.84	0.662	8.25	Tang et al., 2018
PTB7-Th:NA17	0.87	16.48	0.70	10.07	Liu et al., 2016
PTB7-Th:NA18	0.73	20.75	0.63	9.58	Liu F. et al., 2017
PBDB-T:IDTC	0.917	16.56	0.616	9.35	Luo et al., 2017
PBDB-T:IDTO	0.943	16.25	0.654	10.02	Luo et al., 2017
PBDTTT-EFT:IEICO-4F	0.739	22.8	0.594	10.0	Yao et al., 2017a
J52:IEICO-4F	0.734	21.9	0.585	9.4	Yao et al., 2017a

## IDTT based fused-ring acceptors

**Figure 4:**
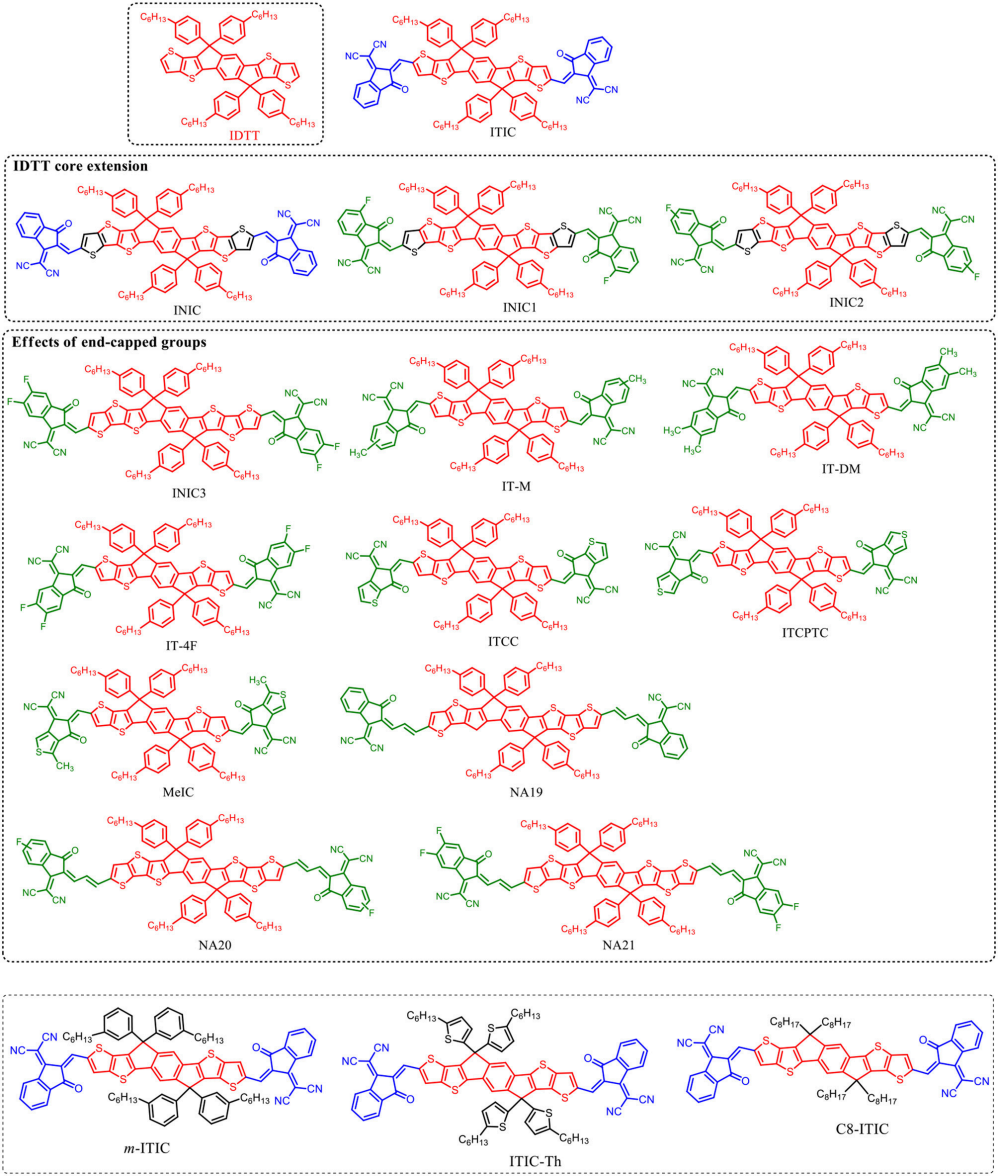
IDTT core based nonfullerene acceptors.

IDTT unit with two additional extended thiophene rings than IDT exhibits excellent planarity (Wong et al., 2006). The pioneering work for IDTT-based nonfullerene acceptor is the development of ITIC which was firstly reported by Lin et al. (2015a) and Zhan et al. (2015). ITIC possesses strong absorption, suitable energy level, good electron transport ability, and good miscibility with various polymer donors. A promising PCE of 6.80% was achieved from the PTB7-Th:ITIC based device (Table 8) (Lin et al., 2015a). Later on, many state-of-the-art ITIC-based PSCs with excellent photovoltaic performance have been reported (Bin et al., 2016; Gao et al., 2016; Qin et al., 2016; Zhao W. et al., 2016; Yang et al., 2017a,b; Xu et al., 2018), and ITIC has been regarded as excellent acceptor for high performance PSCs. To further improve the photovoltaic performance, plenty of molecular design strategies have been carried out to optimize the physicochemical of ITIC. Zhan et al. extended the fused-ring core of ITIC and end-capped with different acceptor unit to develop a series of ITIC derivatives INIC, INIC1, INIC2, and INIC3 (Figure 4) (Dai et al., 2017). Relative to ITIC, INIC with extended conjugated core showed red-shifted absorption spectrum (*E*_g_ = 1.57 eV), slightly higher HOMO level, and slightly lower LUMO level (Table 7). The introduction of fluorine atom onto the end-capped group of INIC effectively red-shifted the absorption and down-shifted the HOMO and LUMO levels (Table 7). The device based on INIC:FTAZ showed a high *V*_oc_ of 0.957 V, with moderate PCE of 7.7%. The fluorination in INIC leads to lower *V*_oc_ values, while significantly improves the FF and *J*_sc_ of the corresponding devices, delivering a higher PCE values (Table 7). The end-capped groups of ITIC show great impact in its photovoltaic performance. Variations of end-capped groups have been conducted onto ITIC to optimize its photovoltaic properties. To up-shift the LUMO level of ITIC without causing too much steric hindrance for intermolecular packing, Hou et al. modulated the LUMO levels of ITIC by incorporating one and two methyls in the end-capped groups (IT-M and IT-DM, Figure 3). Benefited from the weak electron-donating property of methyl, the LUMO levels of IT-M and IT-DM were elevated by 0.04 and 0.09 eV relative to ITIC, respectively (Table 7) (Li et al., 2016). Therefore, higher *V*_oc_ values of 0.94 V and 0.97 V were achieved from the PBDB-T:IT-M and PBDB-T:IT-DM based device, respectively. Encouragingly, a remarkable PCE of 12.05% was realized from the PBDB-T:IT-M based device, with *J*_sc_ = 17.44 mA cm^−2^, and FF = 0.735 (Table 8). One successful molecular optimization on ITIC is the incorporated F-atoms into the end-capping groups to develop IT-4F (Figure 4) (Zhao et al., 2017). Due to the electron-pulling effect of the fluorine atom, IT-4F showed reduced LUMO level, red-shifted absorption spectrum, and enhanced intramolecular charge transfer effects than ITIC. By rational selection of polymer donor with matching energy level, over 13% PCEs have been achieved from IT-4F based PSCs (Cui Y. et al., 2017; Li S. et al., 2018; Zhang et al., 2018). Other end groups engineering such as the replacement of phenyl-fused indanone of ITIC by thienyl-fused indanone as end-groups (ITCC) also affects the electronic properties and enhances intermolecular interactions (Yao et al., 2017b). ITCC possesses larger *E*_g_ of 1.67 eV and up-shifted HOMO and LUMO levels (Table 7) than ITIC. In combination with the improved electron-transport properties and high-lying LUMO level of ITCC, an impressive *V*_oc_ of 1.01 V and a high PCE of 11.4% was achieved from the ITCC based PSC device (Table 8). Changing the orientation of end-capped thiophene of ITCC (ITCPTC, Figure 4) leads to reduced *E*_g_ of 1.58 eV and deeper energy levels (Table 7) (Dongjun et al., 2017). Furthermore, such thiophene-fused ending group can promote the molecular interactions and crystallization compared to ITIC with a benzene-fused end-capped group. The PSCs device using ITCPTC as acceptor and PBT1-EH as donor demonstrated a high PCE of 11.8%, with a remarkable FF of 0.751 (Table 8). Further molecular optimization of ITCPTC is the introduction of methyl onto the thiophene-fused end groups (MeIC, Figure 4) (Luo et al., 2018). Due to the weak electron-donating ability of methyl group, MeIC showed slightly up-shifted LUMO level than ITCPTC and maintained the intramolecular interaction and crystallization. The MeIC-based PSC achieved a high PCE of 12.54%, with a *V*_oc_ of 0.918, a *J*_sc_ of 18.41 mA cm^−2^, and FF of 0.742% (Table 8). Li et al. introduced double bond π-bridges into ITIC to develop three acceptor materials (NA19, NA20, and NA21, as shown in Figure 4) (Li X et al., 2017). The insertion of vinylene π-bridge reduces the *E*_g_, and the fluorine substitution down-shifts the HOMO and LUMO levels of the molecules (Table 7). The PSC device based on J71:NA19 showed a moderate PCE of 7.34%. Significantly enhanced *J*_sc_ of 19.73 mA cm^−2^ was obtained from J71:NA20 based device, with a high PCE of 9.72%. In comparison with the NA19 and NA20 based devices, the devices based on J71:NA21 exhibited the highest PCE of 10.54%, with a notable *J*_sc_ of 20.60 mA cm^−2^.

**Table 7:** Summary of absorption properties and energy levels of IDT core based nonfullerene acceptors shown in Figure 3.

**Acceptor**	**Bandgap [eV]**	**HOMO [eV]**	**LUMO [eV]**
ITIC	1.59	−5.48	−3.83
INIC	1.57	−5.45	−3.88
INIC1	1.56	−5.54	−3.97
INIC2	1.52	−5.52	−3.98
INIC3	1.48	−5.52	−4.02
IT-M	1.60	−5.58	−3.98
IT-DM	1.63	−5.56	−3.93
IT-4F	1.53	−5.66	−4.14
ITCC	1.67	−5.47	−3.76
ITCPTC	1.58	−5.62	−3.96
MeIC	1.58	−5.57	−3.92
NA19	1.40	−5.46	−3.97
NA20	1.37	−5.56	−4.01
NA21	1.35	−5.58	−4.04
*m*-ITIC	1.58	−5.52	−3.82
ITIC-Th	1.60	−5.66	−3.93
C8-ITIC	1.55	−5.63	−3.91

**Table 8:** Summary of photovoltaic properties of the nonfullerene acceptors shown in Figure 4.

**Active layer**	***V*_oc_ [v]**	***J*_sc_ [mA cm^−2^]**	**FF**	**PCE [%]**	**References**
PTB7-Th:ITIC	0.766	10.10	0.551	4.26	Bai et al., 2015b
FTAZ:INIC	0.957	13.51	0.579	7.7	Dai et al., 2017
FTAZ:INIC1	0.929	16.63	0.643	10.1	Dai et al., 2017
FTAZ:INIC2	0.903	17.56	0.668	10.8	Dai et al., 2017
FTAZ:INIC3	0.857	19.44	0.674	11.5	Dai et al., 2017
PBDB-T:ITIC	0.90	16.80	0.742	11.22	Li et al., 2016
PBDB-T:IT-M	0.94	17.44	0.735	12.05	Li et al., 2016
PBDB-T:IT-DM	0.97	16.48	0.706	11.29	Li et al., 2016
PBDB-T-SF:IT-4F	0.88	20.88	0.713	13.10	Zhao et al., 2017
PBDB-T-2Cl:IT-4F	0.86	21.80	0.77	14.4	Zhang et al., 2018
P2:IT-4F	0.90	20.73	0.76	14.2	Li W. et al., 2018
PBDB-T:ITCC	1.01	15.9	0.71	11.4	Yao et al., 2017b
PBT1-EH:ITCPTC	0.95	16.5	0.751	11.8	Dongjun et al., 2017
J71:MeIC	0.918	18.41	0.742	12.54	Luo et al., 2018
J71:NA19	0.89	14.47	0.576	7.34	Li X et al., 2017
J71:NA20	0.84	19.73	0.587	9.72	Li X et al., 2017
J71:NA21	0.81	20.60	0.632	10.54	Li X et al., 2017
J61:ITIC	0.898	17.97	0.655	10.57	Yang et al., 2016
J61:m-ITIC	0.912	18.31	0.755	11.77	Yang et al., 2016
PTB7-Th:ITIC-Th	0.80	15.93	0.680	8.7	Lin et al., 2016b
PDBT-T1:ITIC-Th	0.88	15.80	0.671	9.6	Lin et al., 2016b
PFBDB-T:ITIC	0.95	18.5	0.66	11.71	Fei et al., 2018
PFBDB-T:C8-ITIC	0.94	19.6	0.72	13.2	Fei et al., 2018

Similar to IDT unit, the steric effect of tetrahexylphenyl substituents on the IDTT unit also can reduce intermolecular interactions and prevent the acceptor from forming excessively large crystalline domains when blending with donor material. Thus, the electronic and intramolecular properties can be fine-tuned via the side chains manipulation on IDTT unit. Li et al. developed an analog (*m*-ITIC, Figure 4) by side chain isomerism engineering on the alkyl-phenyl substituents of ITIC (Yang et al., 2016). *m*-ITIC exhibited slightly reduced *E*_g_ and up-shifted LUMO and HOMO levels, while more crystalline and stronger film absorption coefficient than ITIC. In comparison with J61:ITIC based device, overall better photovoltaic performance was realized in J71:*m*-ITIC based device (Table 8). The replacement of phenyl side chains on ITIC by thienyl side chains leads to lower energy levels and increased intermolecular interactions of resulting molecule (ITIC-Th) (Lin et al., 2016b). The enhanced intermolecular interaction of ITIC-Th relative to ITIC should be attributed to the easy polarization of sulfur atom and sulfur-sulfur interaction. A high PCE of 9.6% was obtained from the PDBT-T1:ITIC-Th based device (Table 8). Consider the fact that linear alkyl chains could potentially improve the packing ability and the charge transport mobility of resulting molecules over bulky side chains, Heeney et al. developed an IDTT-based acceptor with linear alkyl side chains (C8-ITIC) (Fei et al., 2018). C8-ITIC showed reduced *E*_g_, higher absorptivity, and increased propensity to crystallize than ITIC. The device based on C8-ITIC recorded an impressive PCE of 13.2%, which is higher than that of ITIC based device (PCE = 11.71%, Table 8).

### Summary and perspective

In summary, we have reviewed the recent progress of IDT and IDTT based nonfullerene acceptors for PSCs. Compared with fullerene acceptors, IDT and IDTT based nonfullerene acceptors offer plenty of molecular design possibilities to tune the physicochemical properties. With the purpose to well-match with the specific donor material, the absorption feature and energy levels of IDT and IDTT based acceptors can be easily and effectively manipulated by rational selection of π-bridge and end-capped groups. Moreover, the intermolecular packing, molecular orientation, as well as crystallinity can be optimized by side-chains engineering to form good morphology with donor materials. Benefiting from the diversification of chemical modification on acceptors and donors, significant progress has been achieved from the nonfullerene acceptors based PSCs. Obviously, the emerging of nonfullerene acceptors brings a bright future for PSCs field. Nevertheless, nonfullerene acceptors still confront challenges. Firstly, although it is straightforward to manipulate the optical absorption and energy level of the IDT and IDTT based molecules, the anisotropic conjugated structures of nonfullerene acceptors make it more complicate to tune the miscibility between donor and acceptor for well-developed morphology. The deep insight into the relationship between molecular structure and photovoltaic should be further exploited. In particularly, various nonfullerene acceptors with different photo-electronic and molecular packing properties have been developed, the rational selection of acceptor material to well-match with polymer donor is essential. Secondly, to further improve the photovoltaic performance, much effort should be devoted to manipulate the energy levels of donor and acceptors for minimizing the energy loss of the devices. Finally, the stability of nonfulleren based device should also be fully investigated.

## Author contributions

The author confirms being the sole contributor of this work and approved it for publication.

### Conflict of interest statement

The author declares that the research was conducted in the absence of any commercial or financial relationships that could be construed as a potential conflict of interest. The reviewer WL and handling Editor declared their shared affiliation.
